# Metastatic Ovarian Serous Adenocarcinoma Clinically Presenting as Inflammatory Breast Cancer

**DOI:** 10.1155/2024/4756335

**Published:** 2024-01-10

**Authors:** Lingling Xian, Rachel Hunter, Emily Smith, Rasha Mohammed, Carlina Madelaire, Guillermo A. Herrera, Rodney E. Shackelford

**Affiliations:** ^1^Department of Pathology, University of South Alabama, 2451 University Hospital Drive, Mobile, AL 36617, USA; ^2^Department of Surgery, University of South Alabama, 1601 Center Street, Suite 2k, Mobile, AL 36604, USA

## Abstract

Metastatic disease to the breast is a rare event, accounting for 0.5-2% of all breast cancers. Outside of metastases from the contralateral breast, malignant ovarian epithelial tumors are the most common origin of these metastases. Here, we present a very rare case of a high-grade ovarian serous adenocarcinoma presenting clinically as inflammatory breast cancer in a 70-year-old woman.

## 1. Introduction

Ovarian cancer is the second most common malignancy after breast cancer in women over forty, is the fifth leading cause of women's cancer-related deaths, and is the most fatal type of gynecologic cancer [1]. Epithelial ovarian malignancies usually metastasize by exfoliation of cells to the omentum, bowel surface, and peritoneal cavity and are usually superficially invasive. Metastatic ovarian cancer dissemination by lymphatic and hematogenous routes is significantly less common [2]. Interestingly, despite being quite rare, primary epithelial ovarian cancers are the most common carcinomas metastatic to the breast [3, 4]. Papillary serous adenocarcinomas are the most frequent ovarian cancers to metastasize to the breast and often present as discreet breast lesions [2–4]. Interestingly, a small subset of these metastases has clinically presented as very rare inflammatory breast cancers, with 12 documented cases [5]. Here, we report an unusual case of a high-grade ovarian serous adenocarcinoma presenting as inflammatory breast cancer.

## 2. Case Report

**Figure 1:**
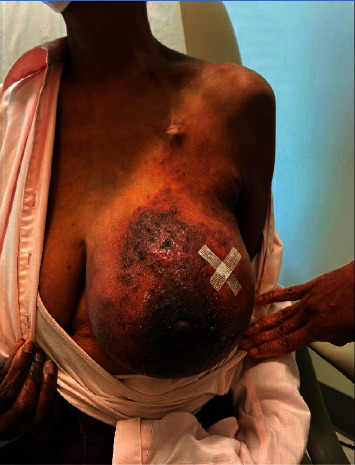
A photograph of the patient's left breast revealing breast swelling, peau d'orange skin changes, and nipple retraction.

A seventy-year-old African American woman presented in March 2023 with a firm, edematous left breast which exhibited diffusely thickened skin with dimpling/pitting and several areas of hyperpigmentation (most notable at 12 o'clock) and mild retraction of the left nipple, with cutaneous dryness and focal blistering. The left axilla had palpable lymphadenopathy. Her symptoms began in November 2022 when the patient started noticing a fullness in her left breast, which she initially attributed to the recent placement of a left IJ port. Her left breast became increasingly more edematous, and she began to experience skin changes, which started to rapidly progress in February 2023 (Figure 1).

The patient had a previous history of an 11.0 cm left ovarian high-grade serous adenocarcinoma, pathologic stage classification pT1c3 pNx pMx, at the time of diagnosis, which had been removed 18 months earlier by total abdominal hysterectomy and bilateral salpingo-oophorectomy. The patient had a family history of ovarian cancer and a CA125 of 2,093.0 U/ml at the time of tumor resection and fell to 127 U/ml postresection. Ovarian tissue biomarker analysis indicated that the tumor was 75% progesterone receptor positive (scored 2+) and was BRCA1/BRCA2, ATM, BRAF, TET2, PPP2R1A, RAD51C, RAD51D, estrogen receptor, and PD-L1 mutation and folate receptor-*α* negative. Additionally, no NTRK1/2/3 fusions were identified. Seven months later, the patient presented with enlarging retroperitoneal lymph nodes, and new upper pelvic soft tissue densities, concerning for disease recurrence/progression, were identified by pelvic computed tomography. Fine needle aspiration analysis determined that the tumor recurrence was immunoreactive for PAX8 and CK7 and immunonegative for WT1 and ER, while MLH1, PMS2, MSH2, and MSH6 expressions were intact.

**Figure 2:**
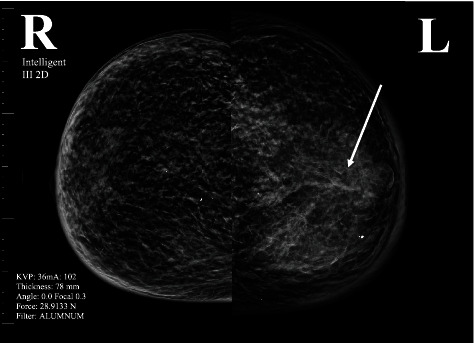
Mammographic comparative analysis of the patient's right and left breasts, with the involved left breast showing scattered fibroglandular densities (arrow) with diffuse increased densities of the left breast with skin thickening. A left breast BI-RADS category 4 score was given, with suspicion for inflammatory carcinoma. The right breast was scored BI-RADS category 1.

**Figure 3:**
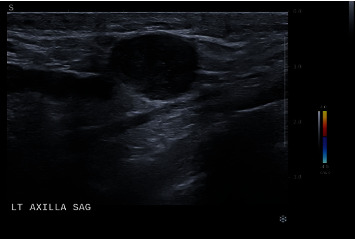
Ultrasound analysis of the patient's left breast which revealed a BI-RADS category 4 irregular, indistinct, and hypoechoic mass measuring 2.0 × 1.5 × 1.2 cm at the 1 : 00 position, 4 cm from the nipple with the left breast.

**Figure 4:**
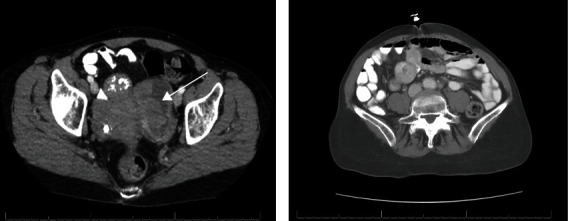
Comparative CT analysis of the patient's abdomen/pelvis before surgery, showing the ovarian mass (a, arrow) and in May 2023, where no abdominal/pelvic recurrence is identified.

A mammogram was performed which revealed diffusely increased scattered left breast fibroglandular densities accompanied by skin thickening. A BI-RADS category 4 score was given, with suspicion for inflammatory carcinoma without calcifications. The right breast was scored as BI-RADS category 1, negative for cancer with calcifications (Figure 2). Two weeks later, an ultrasound was performed which revealed an irregular and indistinct isoechoic mass with posterior shadowing, which measured 2.0 × 1.5 × 1.2 cm at the 1 : 00 position, 4.0 cm from the nipple of the left breast. A BI-RADS category 4 score was given—suspicious abnormality with biopsy recommended (Figure 3). Mammographic analysis of the patient's breasts ten years earlier scored her breasts bilaterally BI-RADS category 1. Interestingly, computed tomographic, X-ray, and magnetic resonance imaging analyses of the patient's chest, brain and head, and pelvis from November 2022 to late May 2023 revealed no pelvic recurrence and no other identifiable metastatic disease besides the patient's left breast (Figure 4).

**Figure 5:**
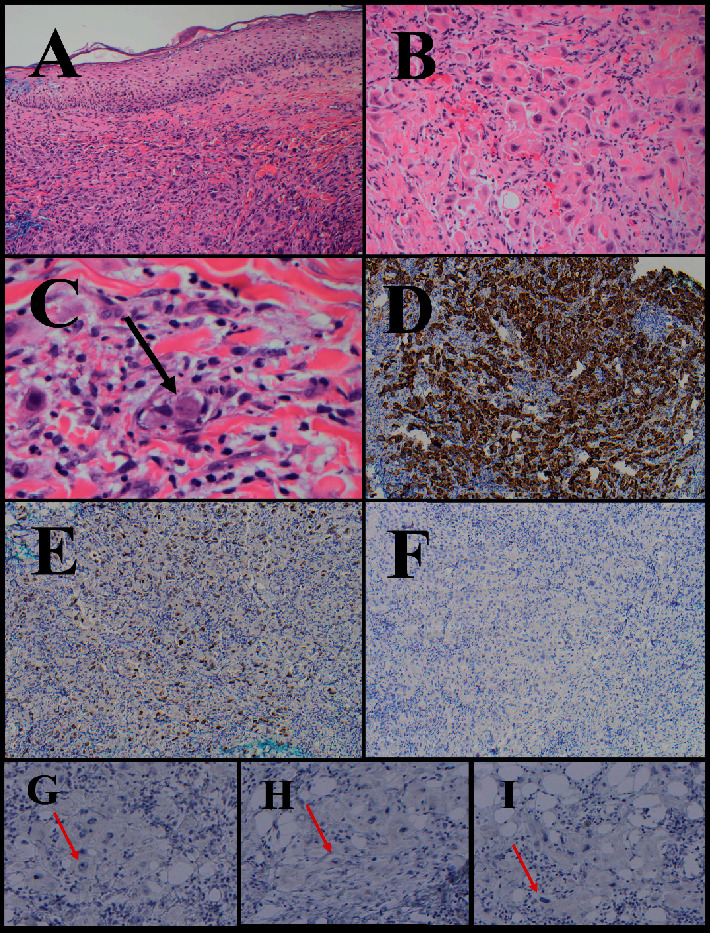
Hematoxylin and eosin stains (H&E) and immunohistochemical analyses of the ovarian serous adenocarcinoma presenting as inflammatory breast cancer. (A) Low-power H&E stain of the left breast, including skin and underlying connective tissue. (B) High-power H&E stain of the left breast dermis showing scattered pleomorphic malignant cells. (C) Very high-power H&E stain of the left breast showing a focus of lymphovascular invasion (arrow). (D) The AE1/AE3 cytokeratin satin reveals infiltrating carcinoma cells within the left breast. PAX8 and GATA-3 immunostains of the left breast showing strong PAX8 nuclear immunoreactivity (E) and GATA-3 nuclear immunonegativity (F), consistent with an ovarian primary. (G–I) The metastatic ovarian tumor cells were also immunonegative for HER2/neu, ER, and PR, also consistent with an ovarian primary (red arrows).

Punch biopsies of the left breast were performed. Examination of hematoxylin and eosin-stained sections of these biopsies revealed that the dermis was infiltrated by numerous highly pleomorphic malignant epithelial cells with large nuclei, prominent nucleoli, and relatively abundant eosinophilic cytoplasm, with focal areas of dermal lymphovascular invasion identified. To further characterize the tumor, immunohistochemical staining for GATA-3, E-cadherin, EMA, PAX8, AE1/AE3, and EMA was performed. The tumor cells showed strong nuclear immunoreactivity for PAX8 and strong membranous immunoreactivity towards AE1/AE3, EMA, and E-cadherin. They were immunonegative for GATA-3, HER2/neu, and the estrogen and progesterone receptors (Figure 5).

As mammographic and ultrasound analyses had revealed a subcutaneous mass, a deeper biopsy was performed. Hematoxylin and eosin-stained sections of the biopsy revealed a similar histologic pattern of numerous pleomorphic malignant epithelial cells with large nuclei, prominent nucleoli, and relatively abundant eosinophilic cytoplasm, identical to those seen in the punch biopsies. Immunohistochemical analysis for AE1/AE3, PAX8, E-cadherin, and GATA-3 showed the same pattern, with immunopositivity seen with the first three stains, accompanied by immunonegativity for GATA-3 (Figure 4). Based on these findings, an ovarian origin for the carcinoma was established. The patient was placed on a clinical trial based on her Acrivon OncoSignature status and treated with Cytoxan, Avastin, and pembrolizumab. However, she developed a high white cell count accompanied by tachycardia, thrombocytopenia, and worsening fatigue. Chemotherapy was postponed due to concerns about possible sepsis. She was admitted and experienced worsening lethargy, mental confusion, and right-sided weakness following a middle cerebral arterial stroke. In late May 2023, she was transferred to inpatient hospice care and died peacefully four days later.

## 3. Discussion

Metastases to the breast are rare and represent only 0.5-2% of all breast cancers [2–5]. The most common metastasis to the breast is from a contralateral primary breast tumor by transthoracic or lymphatic spread [2–5]. Ovarian metastases to the breast represent 0.03-0.6% of these metastatic cancers and, although rare, are the most common nonbreast origin metastatic disease to the breast, with ovarian papillary serous adenocarcinomas being the most common metastases [2–7]. Typically, these patients do not have family histories of breast cancer but may have family histories of ovarian cancer, as the patient presented here did [2–7]. The appearance of the ovarian metastases to the breast can occur concomitantly with the diagnosis, or up to 16 years following diagnosis, with an average time of two years postdiagnosis. In 11-30% of cases, the metastasis is the first manifestation of malignancy, with the patients then surviving being between 13 days and 85 months [2–5]. In the case of our patient, the breast metastasis was identified approximately 570 days after her ovarian cancer diagnosis, and she died 79 days after the initial diagnosis of her ovary to breast metastasis.

In patients with a history of ovarian cancer presenting with breast cancer, the possibility of an ovarian primary must be considered (especially with ovarian serous adenocarcinomas). This is important as primary breast cancers and cancers metastatic to the breast have different treatments and metastatic disease is associated with a poor prognosis. Additionally, the recognition of metastatic disease may aid in avoiding unnecessary surgical procedures [4–7]. Immunohistochemistry is useful in distinguishing to two malignancies. Specifically, WT1, CA125, PAX8, GATA-3, and gross cystic disease fluid protein-15 (GCDFP-15) have been valuable in making this distinction (Table 1). In the patient presented here, PAX8 immunopositivity and GATA-3 immunonegativity were suitable for determining an ovarian origin and excluding a breast primary. Obviously, the initial immunohistochemical characterization of the primary ovarian tumor is part of this analysis. Ovarian cancer metastatic to the breast presenting clinically as inflammatory breast cancer is very rare, with a dozen of documented cases. Interestingly, these rare metastases have a shorter patient survival than do noninflammatory ovary to breast metastases [2–4]. These tumors typically present with rapid breast swelling, peau d'orange skin changes, and nipple retraction subsequent to tumor emboli in the breast dermis, as was seen in the case presented here (Figure 4) [2–4]. Here, we present a 13^th^ example of this rare event. Thus, in patients with ovarian cancer presenting with inflammatory breast cancer, the differential diagnosis should include metastatic ovarian origin.

## Figures and Tables

**Table 1:** Summary of some useful biomarkers to differentiate primary breast cancer from metastatic ovarian cancer [4, 8–13].

IHC marker	Breast cancer	Ovarian cancer
CA125	16% of breast cancers show weak and focal CA125 immunopositivity	~90% of ovarian tumors CA125 positive, with mostly strong and diffuse staining
WT1	WT1 not expressed or very rarely expressed in breast cancer	WT1 expressed in 76% of ovarian cancer and 94% of ovarian serous adenocarcinomas
GCDFP-15	GCDFP-15 expressed in ~71% of metastatic breast cancers	All primary ovarian tumors and metastatic ovarian tumors negative for GCDFP-15
PAX8	PAX8 expressed 0-6.02% of breast tumors, with weak staining seen in some grade III invasive ductal carcinomas	79% or higher PAX8 immunopositivity in ovarian tumors
GATA-3	GATA-3 expressed in ~55-95% of different breast cancer subtypes	~6% GATA-3 expression in different ovarian tumors

## Data Availability

All data has been included within the manuscript.
